# Incidence and Determinants of Newborn Hypoglycemia Among Neonates Hospitalized in Public Hospitals of Wolaita Zone, Southern Ethiopia: A Multicentered Prospective Follow‐Up Study

**DOI:** 10.1155/ijpe/8259158

**Published:** 2026-01-02

**Authors:** Belete Gelaw Walle, Hailu Asmare, Kidst Tadiwos, Banchalem Nega, Bogale Chekole Temere

**Affiliations:** ^1^ Department of Pediatrics and Child Health Nursing, Wolaita Sodo University, Wolaita Sodo, South Ethiopia, Ethiopia, wsu.edu.et; ^2^ Department of Emergency and Critical Care Nursing, Wolaita Sodo University, Wolaita Sodo, South Ethiopia, Ethiopia, wsu.edu.et; ^3^ Department of Midwifery, Wolaita Sodo University, Wolaita Sodo, South Ethiopia, Ethiopia, wsu.edu.et; ^4^ Department of Pediatric and Child Health Nursing, Wolkite University, Wolkite, Central Ethiopia, Ethiopia, wku.edu.et

**Keywords:** Ethiopia, incidence, neonatal hypoglycemia, predictors, Wolaita Zone

## Abstract

**Background:**

Currently, neonatal hypoglycemia is one of the most frequent metabolic disorders and is associated with an increased rate of neonatal morbidity and mortality. Although neonatal hypoglycemia is a common problem among hospitalized neonates, there is limited evidence about its incidence and predictors in Ethiopia, and no studies have been done in our study area. It is critical to consider the incidence and predictors of neonatal hypoglycemia to save neonates′ lives from acute problems, long‐term neurological impairments, and death. This study is aimed at assessing the incidence and predictors of neonatal hypoglycemia among neonates admitted to Wolaita Zone health institutions′ neonatal intensive care units.

**Methods:**

A hospital‐based prospective follow‐up study was conducted on a sample of 785 admitted eligible neonates between November 1, 2023, and June 1, 2024. The eligible neonates were included in the study consecutively. Data were collected using a pretested interview guide and checklist on sociodemographic, obstetric, and neonatal‐related risk factors of hypoglycemia. The collected data were coded, edited, and imported to STATA Version 14 from Kobo toolbox for further analysis. The Kaplan–Meier survival curve was used to determine survival time, and the log‐rank test was used to compare survival times across categorical factors. To identify predictors, both bivariable and multivariable Cox proportional hazard regression models were employed.

**Results:**

Among the 785 neonates included in the final analysis, the overall incidence rate of neonatal hypoglycemia during the follow‐up time was found to be 15.47 per 1000 neonate‐days (95% CI: 12.55, 19.06). Maternal history of bad obstetrics (AHR: 2.26, 95% CI: 1.29, 3.96), gestational diabetes mellitus (AHR: 2.27, 95% CI: 1.27, 4.05), gestational age (AHR: 2.88, 95% CI: 1.35, 6.18), hypothermia (AHR: 2.55, 95% CI: 1.37, 4.77), and newborn type (AHR: 4.31, 95% CI: 1.03, 8.10) were predictors of neonatal hypoglycemia.

**Conclusion:**

In this study, we found a high rate of neonatal hypoglycemia. Therefore, monitoring, identifying, and managing the aforementioned predictors is important to prevent as well as to control neonatal hypoglycemia.

## 1. Background

Neonatal hypoglycemia (NNH) is defined as having a whole blood glucose level of less than 40 mg/dL (2.2 mmol/L) or a plasma glucose level of less than or equal to 45 mg/dL (2.5 mmol/L) [1, 2]. The fetus mainly gets glucose from its mother to maintain the plasma glucose levels. During delivery, the acute interruption of maternal glucose transfer to the fetus needs to support adaptive glucose homeostasis (glycogenolysis and gluconeogenesis). Conversely, some neonates may develop hypoglycemia as they transit from intrauterine to extrauterine life. NNH is one of the most common preventable metabolic disorders related to alterations in metabolism and disruptions in insulin production and function [3]. NNH can be asymptomatic or presented with nonspecific symptoms. In healthy neonates, transient asymptomatic hypoglycemia can be normal throughout the transitional extrauterine life; however, persistent or recurrent hypoglycemia may cause serious neonatal morbidity and mortality [4, 5]. The main clinical presentations of NNH are irritability, shortness of breath, decreased muscle tone, feeding problems, hypothermia, convulsions, or lethargy [6, 7].

NNH affects 10%–20% of normal term babies [8–11], yet it may exceed 60% in high‐risk newborns [12]. From studies conducted in Ethiopia, the prevalence of NNH ranges between 14.89% and 21.2% [13–15]. NNH has both short‐ and long‐term consequences. Short‐term outcomes of NNH commonly include mortality and depressed reflexes, while long‐term outcomes include neurodevelopmental delay (intellectual difficulties, cerebral palsy, developmental delays, and epilepsy), visual impairment, and behavioral problems [5, 6, 16–19]. Multiple gestation, gestational age, birth asphyxia, gestational age for birth weight, pre‐eclampsia/eclampsia, birth weight, maternal toxemia, exchange transfusion, hypothermia, delay in initiation of breast feeding, and sepsis have all been linked to NNH, as demonstrated by studies [1, 12, 15, 20, 21].

The United Nations launched the Sustainable Development Goals (SDGs) in 2015 with the goal of reducing neonatal mortality to less than 12 per 1000 live births by 2030 [22, 23]. Based on the 2019 Mini Ethiopian Demographic and Health Survey, Ethiopia′s neonatal mortality rate was 33 per 1000 live births, which is much higher than the targets for 2025 (22 per 1000) and 2030 (less than 12 per 1000) [23, 24]. To achieve this objective, the Ethiopian government has implemented a variety of methods, including the expansion of emergency obstetric and newborn care services to improve maternal and neonatal health. Despite advances in lowering childhood morbidity and mortality, neonatal mortality remains a major public health concern [25–27].

Identifying predictors of NNH among neonates is critical for reducing risk factors, preventing its onset, and taking appropriate action on time. Even though different interventions have been done, NNH remained high in Ethiopia. Therefore, this prospective follow‐up study was designed to determine the incidence and predictors of NNH. The findings of this study will provide information for policymakers, program managers, and other stakeholders working on neonatal‐related programs to improve neonatal survival in the study area and other areas of Ethiopia.

## 2. Methods

### 2.1. Study Settings, Design, and Period

An institution‐based open prospective follow‐up study was employed from the first of November 2023 to the first of June 2024 in selected hospitals in Southern Ethiopia. Wolaita Sodo is the capital city of the Southern Ethiopia Regional State, which is 378 km away from Addis Ababa, the capital city of Ethiopia. The study was conducted at Wolaita Sodo University Comprehensive Specialized Hospital (WSUCSH), Dubbo Primary Hospital (DPH), and Sodo Christian General Hospital (SCGH). In addition to other healthcare services, these hospitals provide neonatal intensive care services for those seriously ill newborns, including those suffering from hypoglycemia.

### 2.2. Study Participants, Sample Size, and Sampling Procedure

All eligible newborns admitted to the neonatal intensive care units (NICUs) in study areas were the source population, while all newborns admitted to the selected NICUs during the data collection time were the study populations. Abandoned neonates and those whose mothers are not able to communicate were excluded because mothers are the only source of subjective data. Moreover, neonatal readmissions with hypoglycemia presentation were excluded since these are nonincident (prevalent) cases. The minimum required sample size is determined by using the single population proportion formula, a 95% confidence interval, a 3% margin of error, and a 21.2% incidence of NNH from a previous Ethiopian study [14]. Then, the calculated sample size becomes *n* = 713; by adding a 10% nonresponse rate, the adjusted and final sample size to be used for the study is 785. Initially, health institutions with NICU care services at Wolaita Zone were selected purposively. The total average yearly neonatal admission was obtained from each hospital′s health management information system unit and considered to be the study population. Then, the sample size was allocated proportionally to each hospital based on its respective average yearly neonatal admission as of the 2023 report. Finally, all eligible neonates were included based on a consecutive base until saturation of the sample size.

### 2.3. Data Collection Tool and Quality Control

Data were collected prospectively using a pretested and structured interviewer‐administered questionnaire. The questionnaire was developed from related literature [1, 7, 14, 15]. Additionally, a structured checklist adapted from the aforementioned studies was used to abstract data on some maternal (i.e., medical diseases and gestational‐related disorders) and neonatal factors (i.e., sex, weight, age, and place of birth) from maternal interviews and crosschecked with the report as these factors are often recorded in the neonatal chart. Moreover, data collectors underwent random blood sugar (RBS) measurement of neonates using a glucometer at admission across the three hospitals. Data were collected on the exact date (postnatal age) of hypoglycemia occurrence by trained nurses using Kobo Collect. Regarding the subsequent RBS measurement, different patterns of measurement were followed for different groups of neonates. At the time of admission, every newborn in the NICU will receive at least one blood glucose test. Blood glucose levels in high‐risk neonates (neonates with sepsis, asphyxia, preterm, hypothermia, small or large for gestational age, polycythemia, infants of diabetic mothers, respiratory distress, or those with feeding issues) are measured upon admission, then every 3 h for the first 24 h, and then every 6–8 h after that, depending on their clinical condition. To avoid duplicate participation of neonates referred from DPH and SCGH to WSUCSH, a special participant code was written on the referral sheet. All relevant information was collected throughout the follow‐up period on a daily basis. To ensure data quality, data collectors and supervisors at each hospital were given 1 day of training and orientation on the data collection tool and process. Two weeks before the actual data collection, a pretest was conducted at WSUCSH with 5% of the sample size to evaluate the clarity of questions, validity of the instrument, and respondents′ reactions to the questions. Data collectors were nurses from each hospital who had been trained on the fundamental care services of the NICU and were working in the NICU of their respective hospitals. During data collection, data collectors were closely monitored and guided by three MSc in neonatal nursing supervisors for complete and appropriate collection of the data. The primary investigator and supervisors were actively monitoring the whole data collection process and checking the completeness of the data.

### 2.4. Operational Definitions

*A neonate*: Is defined as any child admitted with the age of 28 days or younger [2].

*NNH*: Is defined as RBS level less than 40 mg/dL for any gestational age and postnatal age [2, 28].

*Events*: Eligible neonates who develop hypoglycemia during the follow‐up period.

*Censored*: Neonates who did not develop hypoglycemia and those referred or discharged with parental refusal before an event or free of hypoglycemia during discharge.

*Survival time*: Length of follow‐up from admission of the neonate until the development of NNH.

*Follow-up period*: The time interval between admission to the NICU and the occurrence of an event or censorship.

### 2.5. Data Management and Statistical Analysis

The collected data were coded, cleaned, edited, and analyzed using STATA Version 14 statistical software. Frequencies, proportions, rates, and cross‐tabulations were used to describe the study population in relation to the study variables. Kaplan–Meier curve was used to evaluate the cumulative survival probabilities, and the log‐rank test was used to compare survival curves between groups of the explanatory variables. The proportionality assumption of the Cox proportional hazards regression model was assessed using the Schoenfeld residual test. A bivariable Cox‐proportional hazards regression model was fitted for each explanatory variable, and then variables with *p* value ≤ 0.25 in bivariable analysis were fitted into the Cox‐proportional hazard regression model. Hazard ratios with 95% confidence intervals and *p* values were used to identify statistically significant predictors and measure the strength of association. In the multivariable analysis regression model, variables having *p* value < 0.05 were considered statistically significant predictors of hypoglycemia.

## 3. Results

### 3.1. Baseline Sociodemographic Characteristics

Among the 785 participants included in this study, 501 (63.82%) were male and more than half (443, 56.43%) were from rural areas. The majority of the neonates′ mothers (666, 84.84%) were categorized under the age category 20–34 years old, and 69 (8.79%) were under the 20 years old age category. Among the neonates′ mothers, 109 (13.88%) had no formal education, whereas about 85 (10.83%) had a diploma or above (Table 1).

**Table 1:** Baseline sociodemographic characteristics of study participants in public hospitals of Wolaita Zone, Southern Ethiopia, 2024 (*N* = 785).

**Variables**	**Category**	**Frequency (%)**		
		**Hypoglycemic**	**Nonhypoglycemic**	**Total**
Residence	Urban	49 (6.24%)	293 (37.33%)	342 (43.57%)
	Rural	39 (4.97%)	404 (51.46%)	443 (56.43%)

Maternal age in years	< 20	10 (1.27%)	59 (7.52%)	69 (8.79%)
	20–34	73 (9.30%)	593 (75.54%)	666 (84.84%)
	≥ 35	5 (0.64%)	45 (5.73%)	50 (6.37%)

Age of neonate in hours	≤ 24 h	59 (7.51%)	484 (61.66%)	543 (69.17%)
	25–96 h	18 (2.29%)	150 (19.11%)	168 (21.40%)
	≥ 97 h	11 (1.40)	63 (8.03%)	74 (9.43%)

Sex of neonate	Male	50 (6.37%)	451 (57.45%)	501 (63.82%)
	Female	38 (4.84%)	246 (31.34%)	284 (36.18%)

Marital status	Married	86 (10.96%)	679 (86.50%)	765 (97.46%)
	Single	2 (0.25%)	8 (1.02%)	10 (1.27%)
	Others		10 (1.27%)	10 (1.27%)

Maternal educational status	No formal education	19 (2.42%)	90 (11.46%)	109 (13.88%)
	Primary school	31 (3.95%)	319 (40.64%)	350 (44.59%)
	Secondary school	25 (3.18%)	216 (27.52%)	241 (30.70%)
	Diploma and above	13 (1.66%)	72 (9.17%)	85 (10.83%)

Maternal occupation	Housewife	67 (8.54%)	549 (69.94%)	616 (78.48%)
	Employers	11 (1.40%)	57 (7.26%)	68 (8.66%)
	Others	10 (1.27%)	91 (11.59%)	101 (12.86%)

### 3.2. Maternal‐Related Characteristics of Study Participants

Nearly a third of neonates′ mothers (233, 29.68%) were primipara, and 132 (16.81%) had at least one bad obstetrics history. Most of neonates′ mothers (753, 95.92%) had ANC follow‐up, but only 323 (42.90%) had four or more ANC visits. About one‐third (518, 65.99%) of mothers gave birth at a hospital, while 392 (49.94%) were delivered via spontaneous vaginal delivery. In addition, 489 (62.30%) mothers were taking any medication during their pregnancy or intrapartum period (Table 2).

**Table 2:** Maternal‐related characteristics of study participants in public hospitals of Wolaita Zone, Southern Ethiopia, 2024 (*N* = 785).

**Variables**	**Category**	**Frequency (%)**		
		**Hypoglycemic**	**Nonhypoglycemic**	**Total**
Parity	Primipara	14 (1.78%)	219 (27.90%)	233 (29.68%)
	Multipara	56 (7.13%)	388 (49.43%)	444 (56.56%)
	Grand multipara	18 (2.30%)	90 (11.46%)	108 (13.76%)

ANC visit	Yes	83 (10.57%)	670 (85.35%)	753 (95.92%)
	No	5 (0.64%)	27 (3.44%)	32 (4.08%)

Number of ANC visits	< 4	48 (6.37%)	382 (50.73%)	430 (57.10%)
	≥ 4	35 (4.65%)	288 (38.25%)	323 (42.90%)

History of bad obstetrics	Yes	22 (2.80%)	110 (14.01%)	132 (16.81%)
	No	66 (8.41%)	587 (74.78%)	653 (83.19%)

Gestational diabetes mellitus	Yes	21 (2.68%)	53 (6.75%)	74 (9.43%)
	No	67 (8.53%)	644 (82.04%)	711 (90.57%)

Time of membrane rupture	Premature rupture	21 (2.67%)	104 (13.25%)	125 (15.92%)
	Intrapartum rupture	67 (8.54%)	593 (75.54%)	660 (84.08%)

Duration of membrane rupture in hours	≤ 12	85 (10.83%)	685 (87.26%)	770 (98.09%)
	> 12	3 (0.38%)	12 (1.53%)	15 (1.91%)

Duration of labor in hours	≤24	78 (9.94%)	660 (84.08%)	738 (94.02%)
	> 24	10 (1.27%)	37 (4.71%)	47 (5.98%)

Mode of delivery	Spontaneous vaginal delivery	53 (6.75%)	339 (43.18%)	392 (49.94%)
	Assisted vaginal delivery	24 (3.06%)	166 (21.15%)	190 (24.20%)
	Cesarean section	11 (1.40%)	192 (24.46%)	203 (25.86%)

Place of delivery	Home	6 (0.76%)	25 (3.19%)	31 (3.95%)
	Health center	25 (3.18%)	211 (26.88%)	236 (30.06%)
	Hospital	57 (7.26%)	461 (58.73%)	518 (65.99%)

Meconium‐stained amniotic fluid	Yes	27 (3.44%)	285 (36.31%)	312 (39.75%)
	No	61 (7.77%)	412 (52.48%)	473 (60.25%)

Fetal distress	Yes	38 (4.84%)	42 (5.35%)	80 (10.19%)
	No	50 (6.37%)	655 (83.44%)	705 (89.81%)

Maternal preeclampsia/eclampsia	Yes	15 (1.91%)	81 (10.32%)	96 (12.23%)
	No	73 (9.30%)	616 (78.47%)	689 (87.77%)

Does the mother take any medication during pregnancy and/or intrapartum?	Yes	32 (4.08%)	457 (58.22%)	489 (62.30%)
	No	56 (7.13%)	240 (30.57%)	296 (37.70%)

### 3.3. Neonatal‐Related Characteristics of Study Participants

Of the 785 neonates, more than three‐quarters (637, 81.15%) were term. The majority (710, 90.44%) of patients had an APGAR score of seven and above. Approximately two‐thirds (518, 65.99%) of the neonates were initiated feeding after 1 h, and 95 (12.11%) were hypothermic (Table 3).

**Table 3:** Neonatal‐related characteristics of study participants in public hospitals of Wolaita Zone, Southern Ethiopia, 2024 (*N* = 785).

**Variables**	**Category**	**Frequency (%)**		
		**Hypoglycemic**	**Nonhypoglycemic**	**Total**
Gestational age	Preterm	10 (1.27%)	98 (12.48%)	108 (13.75%)
	Term	76 (9.68%)	561 (71.47%)	637 (81.15%)
	Postterm	2 (0.26%)	38 (4.84%)	40 (5.10%)

Birth weight in grams	< 2500	13 (1.66%)	138 (17.58%)	151 (19.24%)
	≥ 2500	75 (9.55%)	559 (71.21%)	634 (80.76%)

Birth weight for gestational age	Small for gestational age	12 (1.53%)	123 (15.67%)	135 (17.20%)
	Appropriate for gestational age	67 (8.54%)	561 (71.46%)	628 (80.00%)
	Large for gestational age	9 (1.15%)	13 (1.65%)	22 (2.80%)

APGAR score at 5^th^ minutes	< 7	32 (4.08%)	43 (5.48%)	75 (9.56%)
	≥ 7	56 (7.13%)	654 (83.31%)	710 (90.44%)

Newborn type	Singleton	86 (10.96%)	648 (82.55%)	734 (93.50%)
	Twin and more	2 (0.25%)	49 (6.24%)	51 (6.50%)

Initiation time of feeding	Within 1 h	29 (3.69%)	181 (23.06%)	267 (34.01%)
	After 1 h	59 (7.52%)	516 (65.73%)	518 (65.99%)

Hypothermia	Yes	17 (2.17%)	78 (9.94%)	95 (12.11%)
	No	71 (9.04%)	619 (78.85%)	690 (87.89%)

Respiratory rate (breath/minute)	< 30	8 (1.02%)	4 (0.51%)	12 (1.53%)
	30–60	28 (3.57%)	140 (17.83%)	168 (21.40%)
	> 60	52 (6.62%)	553 (70.45%)	605 (77.07%)

Pulse rate (beat/minute)	< 100	6 (0.76%)	15 (1.92%)	21 (2.68%)
	100–150	35 (4.46%)	228 (29.04%)	263 (33.50%)
	> 150	47 (5.99%)	454 (57.83%)	501 (63.82%)

### 3.4. Incidence of NNH

**Figure 1:**
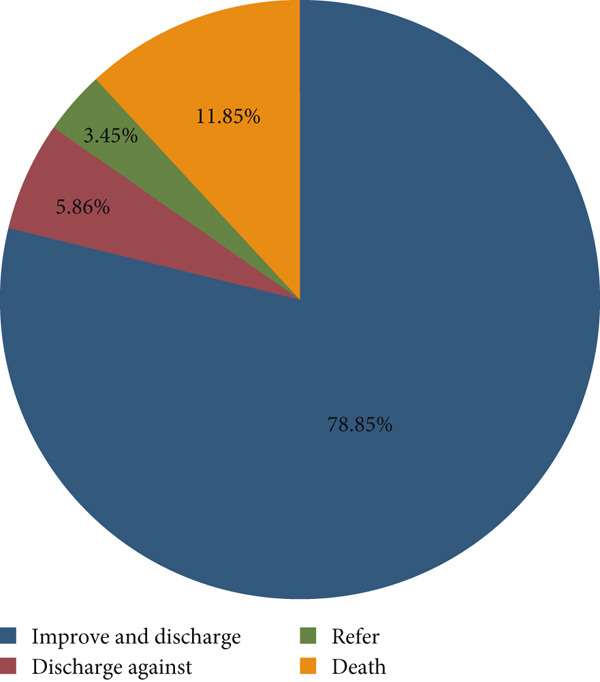
Treatment outcomes of neonates admitted to the NICU in public hospitals of Wolaita Zone, Southern Ethiopia, 2024 (*N* = 785).

**Figure 2:**
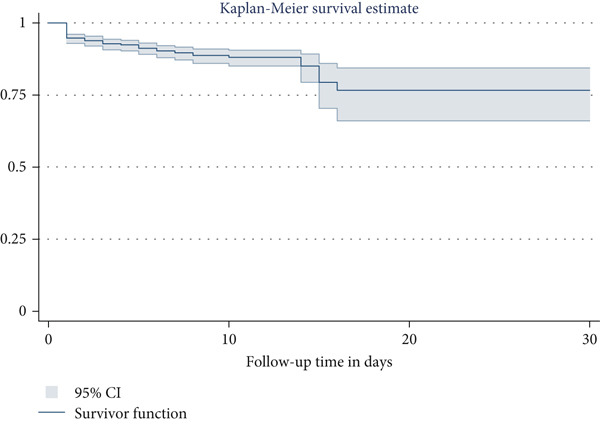
The Kaplan–Meier survival estimate to describe the cumulative probability of neonatal hypoglycemia during follow‐up time in public hospitals of Wolaita Zone, Southern Ethiopia, 2024 (*N* = 785).

The minimum follow‐up time was 1 day with a maximum of 21 days and a total follow‐up time of 5690 person‐days. At the end of the follow‐up period, more than three‐fourths (619, 78.85%) of study participants were discharged with improvement (Figure 1). The overall incidence rate of NNH in all study participants was 15.47 per 1000 person‐days (95% CI: 12.55, 19.06). As shown in Figure 2, the estimated cumulative probability of the survival rate of NNH at the end of the first, second, and third days of follow‐up was 94.78% (95% CI: 92.97, 96.13), 93.89% (95% CI: 91.97, 95.36), and 92.74% (95% CI: 90.69, 94.35), respectively.

### 3.5. Predictors of NNH

In the bivariable Cox‐regression analysis, sex, maternal age, maternal history of bad obstetrics, gestational diabetes mellitus (GDM), fetal distress, mode of delivery, gestational age, birth weight, APGAR score at the 5^th^ minute, hypothermia, initiation time of feeding, and newborn type were predictors selected for the multivariable Cox proportional hazard adjusted model. In the multivariable Cox‐regression analysis, only five variables were found to be statistically significant predictors of hypoglycemia. The results revealed that neonates born to mothers with bad obstetric history were 2.3 times more likely to develop hypoglycemia as compared to those with no history of bad obstetric (AHR: 2.26, 95% CI: 1.29, 3.96). The hazard of developing NNH among neonates who had mothers with GDM was 2.3 times higher (AHR: 2.27, 95% CI: 1.27, 4.05) as compared to their counterparts. Moreover, the hazard of hypoglycemia in preterm neonates was three times higher when compared with those term neonates (AHR: 2.88, 95% CI: 1.35, 6.18). The hazards of hypoglycemia among neonates with hypothermia were 2.5 times higher than those of their nonhypothermic counterparts (AHR: 2.55, 95% CI: 1.37, 4.77). Furthermore, twins and more babies were 4.3 times more likely to develop NNH compared to those singleton babies (AHR: 4.31, 95% CI: 1.03, 8.10) (Table 4).

**Table 4:** The bivariable and multivariable Cox regression analyses of predictors of neonatal hypoglycemia among neonates admitted in public hospitals of Wolaita Zone, Southern Ethiopia, 2024 (*N* = 785).

**Variables**	**Hypoglycemic status**		**CHR (95% CI)**	**AHR (95% CI)**	**p** **value**
	**Event**	**Censored**			
Sex					
Male	50 (6.37%)	451 (57.45%)			
Female	38 (4.84%)	246 (31.34%)	0.74 (0.48, 1.12)	0.75 (0.48, 1.16)	0.20
Maternal age in years					
< 20	10 (1.27%)	59 (7.52%)	1.14 (0.37, 3.47)	1.24 (0.37, 4.16)	0.72
20–34	73 (9.30%)	593 (75.54%)	1.16 (0.47, 2.87)	0.92 (0.34, 2.45)	0.86
≥ 35	5 (0.64%)	45 (5.73%)		1	
Maternal history of bad obstetric					
Yes	22 (2.80%)	110 (14.01%)	1.68 (1.04, 2.72)	2.26 (1.29, 3.96)	0.005 ^∗∗^
No	66 (8.41%)	587 (74.78%)		1	
Gestational diabetes mellitus					
Yes	21 (2.68%)	53 (6.75%)	3.69 (2.28, 5.98)	2.27 (1.27, 4.05)	0.005 ^∗∗^
No	67 (8.53%)	644 (82.04)		1	
Fetal distress					
Yes	38 (4.84%)	42 (5.35%)	5.99 (3.85, 9.34)	1.02 (0.25, 4.20)	0.98
No	50 (6.37%)	655 (83.44%)		1	
Mode of delivery					
Spontaneous vaginal delivery	53 (6.75%)	339 (43.18%)	5.44 (1.67, 17.6)	1.69 (0.39, 7.25)	0.48
Assisted vaginal delivery	24 (3.06%)	166 (21.15%)	1.04 (0.32, 3.32)	0.87 (0.26, 2.88)	0.82
Cesarean section	11 (1.40%)	192 (24.46%)		1	
Gestational age					
Preterm	10 (1.27%)	98 (12.48%)	3.82 (1.90, 7.67)	2.88 (1.35, 6.18)	0.006 ^∗∗^
Term	76 (9.68%)	561 (71.47%)	1.88 (0.81, 4.43)	2.03 (0.86, 4.78)	0.10
Postterm	2 (0.26%)	38 (4.84%)		1	
Birth weight in grams					
< 2500	13 (1.66%)	138 (17.58%)	2.11 (1.35, 3.31)	1.06 (0.57, 1.96)	0.86
≥ 2500	75 (9.55%)	559 (71.21%)		1	
APGAR score at 5^th^ minutes					
< 7	32 (4.08%)	43 (5.48%)	7.02 (4.51, 10.9)	3.44 (0.67, 17.75)	0.14
≥ 7	56 (7.13%)	654 (83.31%)		1	
Hypothermia					
Yes	17 (2.17%)	78 (9.94%)	1.78 (1.05, 3.02)	2.55 (1.37, 4.77)	0.003 ^∗∗^
No	71 (9.04%)	619 (78.85%)		1	
Initiation time of feeding					
Within 1 h	29 (3.69%)	181 (23.06%)		1	
After 1 h	59 (7.52%)	516 (65.73%)	1.84 (1.12, 3.02)	1.24 (0.72, 2.16)	0.44
Newborn type					
Singleton	86 (10.96%)	648 (82.55%)		1	
Twin and more	2 (0.25%)	49 (6.24%)	3.20 (0.79, 13.0)	4.31 (1.03, 8.10)	0.046 ^∗∗^

^∗∗^Significant predictors in the multivariable analysis.

**Figure 3:**
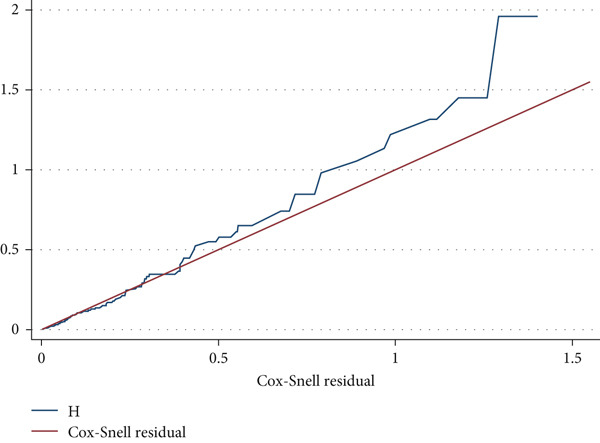
A graph of Cox–Snell residual test showing overall goodness of fit test result among neonates admitted to the NICU in public hospitals of Wolaita Zone, Southern Ethiopia, 2024 (*N* = 785).

The Cox–Snell residual plot was used to evaluate the model′s goodness of fit. The Cox–Snell residuals plot indicates a well‐fitted model, with the hazard function approaching the baseline hazard at 45° (Figure 3). The Schoenfeld residual statistical test (global test) was used to assess the model′s proportionality assumption, which was 0.1414, indicating that it met the assumption criteria.

## 4. Discussion

The main aim of this prospective follow‐up study was to investigate the incidence and predictors of NNH among neonates admitted to public hospital NICUs. The study′s findings may be useful for policymakers and future researchers seeking to reduce neonatal morbidity and mortality associated with hypoglycemia. The findings will help Ethiopia to accomplish its ambitious SDG of reaching an NMR of at least 12/1000 by 2030, as well as in the research area specifically. In fact, reducing the incidence of NNH is crucial for maintaining and enhancing quality of life.

The overall incidence rate of NNH during the follow‐up time was found to be 15.47 per 1000 person‐days (95% CI: 12.55, 19.06), which is comparable with the studies from India [29–31], Kenya [32], and the study done at Black Lion Specialized Hospital in Ethiopia [13]. However, the finding of this study was lower than studies done in Ethiopia [15, 33, 34], Nigeria [35, 36], Bangladesh, and the Netherlands [37]. On the other hand, the finding was higher than studies done in Egypt [38], Ghana [39], Northern Uganda [40], the United States [41], Nigeria [42], Madagascar [43], and India [44]. The possible reason for this discrepancy might be due to variations in the follow‐up period, study sites, study design, sample size, method used to assess neonates′ blood glucose concentrations or diagnostic criteria, and sociodemographic characteristics of study participants. Moreover, the level of NICU, availability of advanced equipment, and quality of service are different between developed and developing nations [45]. These factors individually and collectively could affect the incidence of NNH.

The current study explored predictors of NNH among study participants. In this follow‐up study, maternal bad obstetric history (i.e., IUFD, abortion, still birth, or neonatal death) was significantly associated with NNH. As a result, neonates born to mothers who had a history of bad obstetric history were more likely to develop hypoglycemia as compared to their counterparts. Maternal history of IUFD, abortion, stillbirth, or neonatal death may suggest the existence of chronic maternal conditions such as diabetes, hypertension, or other systemic diseases. These underlying maternal conditions can influence fetal development and glucose metabolism, potentially leading to NNH in subsequent pregnancies [46, 47].

This research revealed a significant correlation between the incidence of NNH and the presence of maternal GDM. Neonates born from mothers with GDM were more likely to have NNH than neonates born from mothers without GDM. The finding of this study was supported by prior studies done in Ethiopia, Kenya, China, and India [14, 21, 28, 32, 34]. Following maternal hyperglycemia in GDM, more glucose crosses the placenta, raising the fetus′s blood glucose levels as well. To control the elevated blood glucose levels, the fetal pancreas produces more insulin. When the maternal glucose supply is cut off after birth, the newborn may still produce high levels of insulin, resulting in a sudden decline in blood glucose levels [46, 48]. Additionally, neonates born to mothers with GDM may have less glycogen stores in the liver, which may affect their capacity to maintain normal blood sugar levels after birth, especially if they do not feed well immediately after delivery [49]. NNH can occur from insulin overproduction and insufficient glycogen storage during and after the transition period [48, 50].

Additionally, prematurity was significantly associated with NNH. This follow‐up study showed that preterm neonates were more likely to develop hypoglycemia than their term counterparts. The finding is in line with studies undertaken in Bangladesh [51], Indonesia [52], Nigeria [42, 53], Kenya [32], and Ethiopia [14, 33]. This could be explained by the scientific data that in preterm neonates have immature liver and endocrine systems, which can limit their ability to regulate blood glucose levels efficiently [46, 47]. They have less glycogen stores in the liver than full‐term newborns. This implies they have fewer reserve to draw upon when their blood glucose levels fall. Preterm newborns have trouble feeding, whether due to a weak suckling reflex or difficulties with feeding coordination, which can contribute to insufficient glucose intake. However, these neonates have higher metabolic demands because of their rapid growth and development, which might lead to a higher risk of hypoglycemia if their intake does not satisfy their needs [54].

Moreover, this study demonstrated that neonates with hypothermia were more likely to experience hypoglycemia than neonates without hypothermia. The finding is consistent with studies conducted in the United States [55], Bangladesh [51], Nigeria [36], and Ethiopia [15, 33]. This might be due to the fact that hypothermia increases metabolic rate to generate heat, which requires more energy and, as a result, can lower blood glucose levels faster. It can reduce the liver′s ability to produce glucose from noncarbohydrate sources (gluconeogenesis). This means that even if the baby′s glycogen stores are not entirely depleted, the ability to produce new glucose is impaired. In general, hypothermia imposes additional metabolic demands, and when these needs exceed the available glucose stores or the ability to regulate blood sugar levels, the newborn can develop hypoglycemia [42, 56].

Finally, this study found that multiple gestations (twins or more neonates) were more likely to develop hypoglycemia than singleton neonates. This finding is consistent with studies conducted in Italy [57] and the Netherlands [37]. This might be due to preterm birth, lower birth weight, or maternal complications related to multiple pregnancies.

To address the identified risk factors, it is imperative to improve antenatal screening programs for GDM detection and management, increase the capacity of early glucose monitoring among high‐risk neonates, encourage early initiation of breastfeeding, prevent hypothermia through kangaroo care, and engage families on maternal and neonatal health. Moreover, targeted interventions that address maternal health inequities, improve referral systems, and integrate evidence‐based neonatal care practices are essential.

### 4.1. Limitations and Strengths of the Study

This study has both limitations and strengths. In this study, the impact of training, supplies, and equipment was not explored, and although similar measurement procedures and glucometers were used across hospitals, minor interhospital variability and the inherent limitations of point‐of‐care glucose testing could have introduced small measurement differences or misclassification. The other significant limitation of this study is the absence of long‐term neurodevelopmental follow‐up to explore the long‐term impacts of NNH. Additionally, as the study was done at the hospital level, it may not address the general community. Despite these limitations, the study has a number of strengths. This follow‐up study was conducted prospectively in multiple settings. As a result, we were able to include a range of sociodemographic, obstetric, and neonatal‐related variables that were very important to determine NNH.

## 5. Conclusion

In this follow‐up study, we found a high rate of NNH. Maternal history of bad obstetric, GDM, gestational age, hypothermia, and newborn type were predictors significantly associated with NNH. Based on our findings, we strongly recommend that special emphasis shall be given to premature and hypothermic neonates admitted to NICU. Health education about maternal bad obstetric history as well as GDM shall be given to the mothers during ANC and postnatal follow‐up care. Multiple gestations should get special attention since they are associated with the occurrence of NNH. Finally, further long‐term follow‐up research is required to identify the outcomes and explore the impact of factors that were not addressed.

**Nomenclature**
AHR: adjusted hazard ratio
ANC: antenatal care
CHR: crude hazard ratio
CI: confidence interval
GDM: gestational diabetes mellitus
NICU: neonatal intensive care unit

## Ethics Statement

Ethical clearance was obtained from the Institutional Review Committee of Wolaita Sodo University, College of Health Science and Medicine. Following the approval, an official letter of cooperation was submitted to selected health institutions. After the explanation of the study, informed, verbal voluntary consent was obtained from each participant (mother). As the study was conducted through a face‐to‐face interview and chart review, the individual participants were not exposed to any harm. Moreover, the mothers were informed that the information they gave would be treated with complete confidentiality and would not cause any harm. Data collectors were applying infection prevention techniques like proper hand washing, gloving, and alcohol rubbing of instruments before touching neonates for the nonmaleficent sake of the study. Neonates who were found to be hypoglycemic and hypothermic were managed as per the NICU management protocol.

## Consent

The authors have nothing to report.

## Disclosure

All authors have read and approved the final version of the manuscript.

## Conflicts of Interest

The authors declare no conflicts of interest.

## Author Contributions

B.G.W.: conception of research protocol, study design, literature review, statistical analysis, data interpretation, coordinating the data collection process, and developing the first draft of the manuscript. H.A. and K.T.: approval of the proposal with revisions, data analysis, interpretation, and manuscript write‐up. B.N. and B.C.T.: reinterpreted the reanalysis and revised the manuscript critically.

## Funding

This study was funded by the Wolaita Sodo University (WSU) (41/38/1581).

## Data Availability

All the data supporting the study findings are within the manuscript. The additional detailed raw datasets used during this study are available from the corresponding author upon reasonable request.
